# Knowledge, Attitudes, and Practices on Tick-Borne Encephalitis Virus and Tick-Borne Diseases within Professionally Tick-Exposed Persons, Health Care Workers, and General Population in Serbia: A Questionnaire-Based Study

**DOI:** 10.3390/ijerph19020867

**Published:** 2022-01-13

**Authors:** Ana Vasić, Jovana Bjekić, Gorana Veinović, Darko Mihaljica, Ratko Sukara, Jasmina Poluga, Saša R. Filipović, Snežana Tomanović

**Affiliations:** 1 Institute for Medical Research, National Institute of Republic of Serbia, University of Belgrade, Dr Subotića 4, 11000 Belgrade, Serbia; ana.vasic@imi.bg.ac.rs (A.V.); jovana.bjekic@imi.bg.ac.rs (J.B.); gorana.veinovic@imi.bg.ac.rs (G.V.); darko.mihaljica@imi.bg.ac.rs (D.M.); ratko.sukara@imi.bg.ac.rs (R.S.); sasa.filipovic@imi.bg.ac.rs (S.R.F.); 2 Clinic for Infectious and Tropical Diseases, University Clinical Centre of Serbia, Bulevar Oslobođenja 16, 11000 Belgrade, Serbia; poluga@eunet.rs; 3 Faculty of Medicine, University of Belgrade, Dr Subotića 8, 11000 Belgrade, Serbia

**Keywords:** tick-borne encephalitis virus, tick-borne diseases, professionally tick-exposed persons, health care workers, general population, Serbia

## Abstract

This study assessed the level of knowledge, attitudes, and practices (KAP) regarding tick-borne encephalitis virus (TBEV) and tick-borne diseases (TBDs) among different groups of people in Serbia. Professionally tick-exposed persons (PTEPs), health care workers (HCWs), and the general population (GP) were subjected to an anonymous, voluntary, online questionnaire using Microsoft Forms. A total of 663 questionnaire responses were collected (February–March 2021), while 642 were included in the analysis. The significant difference in knowledge in TBDs existed between GP and PTEPs, and HCWs (*p* < 0.001). The perception of risk-to-tick exposure and TBDs was generally high (42.4 (95% CI: 33.6–51.2) within GP, 44.9 (95% CI: 35.8–53.9) within PTEPs and 46.2 (95% CI: 38.0–54.5) within HCWs), while fear was low (13.7 (95% CI: 7.9–19.5) within GP, 12.6 (95% CI: 7.3–19.9) within PTEPs, and 13.5 (95% CI: 7.4–19.5) within HCWs). Protective practices differed across groups (F (2639) = 12.920, *p* < 0.001, η^2^ = 0.039), with both PTEPs (t = 3.621, Cohen d = 0.332, *p* < 0.001) and HCWs (t = 4.644, Cohen d = 0.468, *p* < 0.001) adhering to more protective practices than the GP, without differences between PTEPs and HCWs (t = 1.256, Cohen d = 0.137, *p* = 0.421). Further education about TBDs in Serbia is required and critical points were identified in this study.

## 1. Introduction

Ticks are the second most important vector group in the world following mosquitoes [1], which transmit pathogens (tick-borne pathogens (TBPs)) of different origins: viral, bacterial, protozoal. In various parts of Europe, tick-borne diseases (TBDs) are the most spread and prevalent of all vector-borne diseases [2], causing significant economic losses in the treatment of patients (estimated to be EUR 19.3 million in the Netherlands [3] and EUR 80 million in Germany [4]). A flavivirus, tick-borne encephalitis virus (TBEV) incidence has been increasing in Europe, causing a high disease burden of tick-borne encephalitis (TBE) and costs for health care and society [5,6,7]. The European Centre for Disease Prevention and Control (ECDC) included TBE in the list of notifiable diseases in the European Union (EU) in September 2012, as a disease with available preventive measures but causing significant morbidity [8]. In central European countries (e.g., Austria, Switzerland, Slovenia), TBEV is endemic, and the vaccination of inhabitants has been conducted for many years. Nevertheless, vaccination coverage is low in most endemic countries and inefficient to adequately reduce the TBE burden [9].

The clinical appearance of TBE in humans is characterized by the biphasic occurrence of symptoms in some infected people resulting in severe neurological symptoms (meningitis, meningoencephalitis). Treatment for TBE is symptomatic and without specific therapy [10], the overall mortality is estimated to be 1%, and survivors face long recovery processes, neurological sequelae, and a decreased quality of life [11].

Three subtypes of TBEV were initially known to circulate depending on their geographical distribution: the European (TBEV-EU), the Far Eastern (TBEV-FE), and Siberian (TBEV-Sib). Recently, new strains were isolated and considered as new subtypes in Russia [12,13] and China [14]. Even though the food-borne transmission of TBE (via unpasteurized milk or dairy products) is an often-encountered infection route in Europe [15], transmission via tick bite represents the primary route of infection [5]. It is known that 22 tick species can carry TBEV [16]. In Europe, the most important and widespread vector is *Ixodes ricinus*, while in Asia, it is *Ixodes persulcatus* [16]. The role of other tick species in TBEV transmission in nature remains mostly unknown. New data suggest a the role for *Dermacentor reticulatus* in the natural cycle of TBEV in Europe [17,18].

In Serbia, the presence of TBE was firstly noted based on serological evidence in healthy humans from 1962 to 1969, when the presence of TBEV-specific antibodies by hemagglutination inhibition test was noted in the areas of Srem (1.1%), central Serbia (2%), eastern Serbia (3.6%), Belgrade (7.3%), Banat (8.4%), western Serbia (19.4%), Kosovo (37.8%) and Sandžak (52.6%). In 1972, the TBEV virus was isolated from a tick from Sandžak [19]. A recent study confirmed the presence of TBEV-specific antibodies in healthy humans in the South Bačka area (7.9%), while in southern Serbia, there were no positives among the tested [20]. Moreover, TBEV seroprevalence by ELISA test without confirmation test was 13.27% in tick-bitten individuals compared to 4% in healthy blood donors, while none developed clinical illness [21]. Nevertheless, TBE clinical disease is present in humans in Serbia: a series of clinical cases occurred in 2017, implying that the TBEV affects the human population [22]. However, not only humans are exposed to TBEV in Serbia. The seroprevalence was found to be 17.5% in dogs, 5% in horses, 12.5% in wild boars, 2.5% in cattle, and 2.5% in roe deer [23]. In the same study, the molecular evidence of TBEV was found in two *I. ricinus* ticks, confirming the presence of the western European TBEV subtype in northern Serbia (Fruška Gora mountain) and Belgrade [23]. The obligatory declaration of TBE to health authorities has existed since 2004, but TBE in Serbia is not considered an endemic disease [24].

Vector-borne diseases are sensitive to climate change and global warming; there is increasing data suggesting that many of these diseases will expand their geographical range and impact human health [25]. Vector-borne diseases are recognized as one of the links between climate change, human health, and occupational safety health [26]. Exposure to tick bites poses a health risk for humans working in the natural habitat. Therefore, the risk of TBD occurrence is an increasing problem for occupational health in Europe.

While populations especially vulnerable to TBE include professionally tick-exposed persons, it is health care workers that are responsible for performing the prophylaxis, diagnostics, and therapy of tick-borne diseases [27,28]. The occurrence of TBE is more frequent in risk groups such as forestry and agriculture workers, hunters, hikers, ramblers, mushroom and berries collectors [29]. Usually, TBE control efforts in non-endemic countries, such as Serbia, should be directed to, e.g., monitoring in vectors, a prophylaxis approach to persons at risk in non-endemic areas and travelers to endemic areas [10], as well as education on tick bite prevention [30]. Another problem in TBE monitoring is the frequent lack of etiological diagnosis of TBE in patients, as its certain diagnosis often does not change the treatment of the patients or the outcome of the disease, and thus base their diagnosis on the definitions of probable or suspected cases [31]. Etiological diagnostics are not being regularly performed and awareness of the disease is generally low in many countries including Serbia [9].

The Health Belief Model (HBM) is a psychological health behavior change model which explains and predicts health-related behaviors, particularly in regard to the uptake of health services [32]. Based on particular constructs such as the perceived susceptibility, severity, benefits, barriers as well as action and self-efficacy of an individual, this theory can be used for the estimation of people’s response toward ticks and TBDs [33]. This model is the extension of expectancy-value theory and puts forward perceived susceptibility as one of the main psychological drivers of one’s health-related behavior. In the case of TBE, perceived susceptibility can be conceptualized as perceived risk from ticks and TBDs. Complementary to the HBM, which focuses on cognition-to-behavior dynamics, emotion-based models (e.g., protection motivation theory [34]) put forward fear as a key factor for predicting health-related behaviors. Both the risk of ticks and the fear of ticks are in interplay with the knowledge regarding ticks and TBDs, as this has been the case for other diseases including COVID-19 [35].

As the incidence of TBE in Serbia increases, in addition to the necessary serological and entomological studies as well as etiological diagnostics of TBDs, it is also necessary to raise awareness regarding the prevention of tick-bites and TBDs. This study aimed to estimate and compare the level of knowledge, attitudes, and practices of professionally tick-exposed persons (PTEPs), health care workers (HCWs), and the general population (GP) in Serbia towards TBE and TBDs. Moreover, this study was performed to provide a baseline in the future education of selected groups and to propose public health strategies in the field of TBDs.

## 2. Materials and Methods

An anonymous, voluntary, online questionnaire (Supplementary Material S1) was designed and distributed from 24 February to 24 March 2021 using Microsoft Forms (Microsoft Corporation, Redmond, WA, USA). The participants were recruited via professional networks (for professionally tick-exposed persons and health care workers) and via social media coverage for the general population. The questionnaire contained questions differentiating three groups: professionally tick-exposed persons (PTEPs); health care workers (HCWs); and the general population (GP). The survey was conducted in the Serbian language. The preliminary check on the statistical relevance of the results was performed on the first 100 filled questionnaires later included in the final results of the study. This study was approved by the Ethical Board of the Institute for Medical Research, National Institute of Republic of Serbia, the University of Belgrade (permission number EO137/2021).

The “professionally tick-exposed persons” (PTEPs) are defined as persons who, by the nature of their work, regularly stay in an environment in which they are in contact with ticks (e.g., forestry workers, veterinary professionals, hunters—they were specifically recruited for the study via their respective associations). The “health care workers” (HCWs) were defined as persons employed in the health care sector in Serbia regardless of their educational level (e.g., nurses, medical doctors; in primary, secondary, and tertiary level; in the public and private sectors), while all other participants were referred to as the “general population” (GP).

The questionnaire included 4 sections:

1. Entity characteristics: a place of residence (free entry); age (free entry); sex; educational level; having children under the age of 18; having dog(s); being a professionally tick-exposed person; experiencing a tick bite in a lifetime; having TBD(s); having a family member diagnosed with TBD(s); being a health care worker; and employment in primary health care in Serbia.
2. Knowledge and opinion towards ticks and TBPs: familiarity with the term “arbovirus”; endemic area; TBE; frequency of TBDs in Serbia; number of developmental stages of ticks; time frame in which ticks could transmit a TBEV; the possible lethal outcome of TBE; registered cases of TBE in Serbia; availability of the vaccine against TBE in the world; the occurrence of TBE in Europe; whether ticks can transmit causative agents of Lyme borreliosis; West Nile fever (WNV); Crimean–Congo hemorrhagic fever (CCHF); whether only one tick species could transmit tick-borne pathogens; whether the tick should be removed from the skin by use of medical oil, tweezer and in whole; whether every tick is infected with TBP(s); and whether the causative agent of TBE is a bacterium. Each of the 19 items was presented as a binary forced-choice (Yes/No) response to statements with the “I don’t know” option.
3. Attitudes towards ticks and TBDs through the estimation of (a) risk and risk perception from ticks and TBDs: entering risky situations in general, being in nature and long-term being in nature as a risk of tick-bite, whether a tick bite could lead to the development of TBD(s), whether TBE could be deadly, whether the risk of TBE in Europe is increasing, whether vaccination could prevent TBE, walking through or on fields/woods/river banks/paths as a risk of a tick bite, whether ticks can pass from dog to human, whether tick bites are significant cause of concern; and (b) fear of ticks and TBDs: fear of ticks, TBDs, whether the fear of tick bite affects one’s desire towards spending time in nature, whether the fear of ticks repels people from grass areas, whether the fear of ticks prevents children playing in grass, whether the fear of ticks and TBPs influences everyday life, and whether the fear of TBE influences everyday life. The items were assessed through a 5-point Likert type scale (1—strongly disagree; 5—strongly agree).
4. Protective behavior towards ticks and TBDs: use of repellents; wearing long sleeves; white clothes; socks; avoiding high grass; checking one’s skin after being in nature; protection against ticks; using tick repellent on dogs; vaccination against TBE and testing to TBDs; informing oneself through a chosen medical doctor with regard to what vaccinations are needed before trips abroad; protection in the workplace; acceptance of vaccination towards TBPs in general; informing about TBDs and tick control programs; and whether having a dog increases the risk of acquiring a TBD. Items were presented as a binary forced choice (Yes/No) response to statements in addition to the “I don’t know” option.

The statistical analysis was performed in JASP 0.14.1.0. (University of Amsterdam, The Netherlands). We conducted descriptive statistical analysis to learn about the properties among tested groups. Furthermore, frequency tables were employed for analyzing categorical data and screening for entry errors. For the assessment of the internal consistency of the questionnaire sections, Cronbach’s alpha coefficients were calculated. A knowledge score was calculated as a summary score of the correct answers. Risk and risk perception as well as fear and fear perception scores were calculated as summary scores after recoding the inverse items. Therefore, the theoretical range for risk was 13–65 points while for fear it ranged between 7 and 35 points.

To test the differences between groups, one-way ANOVA for independent samples—with between-subject factor group (3 levels: PTEPs/GP/HCWs), and questionnaire responses/summary scores as dependent variables—were performed. Between-groups post hoc comparisons (*t*-tests) were also performed using Holm correction for multiple comparisons. As the measure of the effects’ size, Cohen d and eta square (η^2^) were used. The significance threshold was set at *p* < 0.05 across all analyses.

## 3. Results

A total of 663 completed questionnaires were collected during the period of February–March 2021. After the preliminary data analysis and exclusion of 21 questionnaires based on the declaration of a place of residence outside Serbia, 642 questionaries were further analyzed. The participants belonged to three groups: PTEPs (*n* = 199), HCWs (*n* = 147) and GP (*n* = 296). Among the tested persons, the mean age of the participants was 42.9 (95% CI: 30.3–55.5) years in PTEPs, 43.1 (95% CI: 30.6–55.7) in HCWs, and 39.7 (95% CI: 28.4–50.9) in GP. In this study, 240 females, 400 males, and 2 persons declared as other participated. The educational level of the questioned persons was as follows: high school education (*n* = 66); faculty graduation level (*n* = 252); postgraduate specialization level (*n* = 53); postgraduate master studies (*n* = 103); Ph.D. level (*n* = 168). Among the participants, 44.2% (95% CI: 43.7–44.7) had a dog as an additional tick exposure risk factor. Additionally, 42.8% (95% CI: 42.8–42.9) of participants had children, which was also recognized as a driving factor towards spending time in ticks’ natural habitats. The relevant characteristics of each tested group are summarized in Table 1.

### 3.1. Knowledge about Ticks and TBDs in Serbia

**Figure 1:**
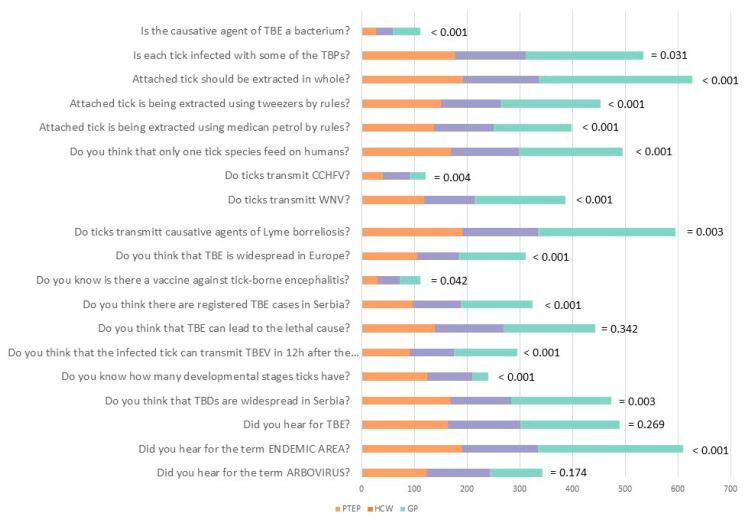
Questions on the knowledge and opinion towards ticks and tick-borne diseases and the answers of the participants from the three different groups.

The questionnaire included 19 questions that assessed participants’ level of knowledge among three groups (Figure 1). Overall, participants correctly answered in the highest percent the questions concerning the endemic area (95.0%, (95% CI: 93.0–96.6)), the transmission of Lyme borreliosis by ticks (92.8%, (95% CI: 90.6–94.7)) and necessity to extract the attached tick in whole (97.8%, (95% CI: 96.4–98.8)). The lowest correct answer rate was noted in questions considering TBE vaccination (17.4%, (95% CI: 14.6–20.6)]), whether ticks could transmit CCHF (19.0%, (95% CI: 16.0–22.3)) and whether they could be the causative agent of TBE (17.3%, (95% CI: 14.4–20.4)). ANOVA revealed significant group differences (F_(2,639)_ = 51.424, *p* < 0.001, η^2^ = 0.139). Namely, the GP group had lower scores than both HCWs (t_(3)_ = 9.606, Cohen d = 0.946, *p* < 0.001) and PTEPs (t_(3)_ = 6.534, Cohen d = 0.578, *p* < 0.001), and HCWs showed significantly higher knowledge levels than PTEPs (t_(3)_ = 3.405, Cohen d = 0.407, *p* = 0.002). At the individual items level, the differences between GP and HCWs were the most pronounced regarding the knowledge of endemic area (t_(2)_ = 2.619, *p* = 0.027); TBE transmission after tick-bite (t_(2)_ = 3.322, *p* = 0.003); the occurrence of TBE in Serbia (t_(2)_ = 3.320, *p* = 0.003)); and whether tick extraction by tweezers is correct (t_(2)_ = 2.930, *p* = 0.011). All group differences are shown in Supplementary material S2. 

### 3.2. Attitudes towards TBD

**Figure 2:**
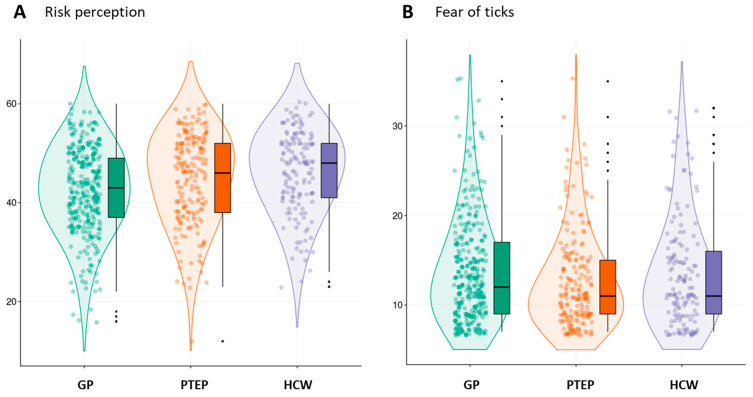
(**A**) The perception of risk of ticks, tick-borne encephalitis, and tick-borne diseases; and (**B**) fear of ticks, tick-borne encephalitis, and tick-borne diseases in the three tested groups: general population (GP), professionally tick-exposed persons (PTEPs), and health care workers (HCWs).

The attitudes included risk-of-ticks perception and fear of ticks, with the questionnaire showing satisfactory internal constancy (Cronbach’s α > 0.83) and the questions tapping into the subjective evaluation of risk and risk perception in connection with ticks and TBPs and the estimation of the fear of tick bite, TBE and TBDs. The perception of risk of tick exposure and TBDs was high in all three groups (M = 42.38 (33.60–51.16) for GP, M = 44.87 (35.81–53.93) for PTEPs and M = 46.23 (37.96–54.49) for HCWs). However, some group differences were still observed (F_(2,639)_ = 10.807, *p* < 0.001, η^2^ = 0.033), with both PTEPs and HCWs showing higher levels of perceived risk than the GP group (t_(2)_ = 3.104, *p* = 0.002; t_(2)_ = 4.356, *p* < 0.001, respectively). The parameters of fear-of-ticks and TBPs remained low and without statistical differences between the tested groups (M = 13.74 (7.95–19.53) for GP, M = 12.57 (7.29–19.86) for PTEPs and M = 13.48 (7.45–19.51) for HCWs). Fear of ticks did not reach statistical significance across all groups F_(2,639)_ = 2.575, *p* = 0.077); however, planned simple contrast showed that PTEPs had a slightly higher fear than the GP group (t_(2)_ = 2.234, *p* = 0.026), with no differences between the HCWs and GP (t_(2)_ = 0.447, *p* = 0.655) (Figure 2).

### 3.3. Protective Practices

Protective practices also differed across groups (F_(2,639)_ = 12.920, *p* < 0.001, η^2^ = 0.039), with both PTEPs (t_(2)_ = 3.621, Cohen d = 0.332, *p* < 0.001) and HCWs (t_(2)_ = 4.644, Cohen d = 0.468, *p* < 0.001) adhering to more protective practices than the GP group, without a significant difference between PTEPs and HCWs (t_(2)_ = 1.256, Cohen d = 0.137, *p* = 0.421). In further detail, only 90 participants declared that they wear white clothes when in nature, while approximately half (*n* = 323) wear long sleeves. The majority of participants check their skin for ticks after being in nature (*n* = 548). Dog owners mostly think that the protection of pets against ticks helps their protection against ticks (*n* = 376), while the majority (*n* = 441) think that having a dog does not increase the risk of tick acquisition. Among participants, 379 informed themselves before traveling to a foreign country about the diseases present there, while only 193 informed themselves about the necessary vaccinations before traveling. Only 53 participants replied positively to the question of whether they asked their physician about the protection from ticks during work hours, with similar numbers of positive answers in all three groups (PTEPs = 17, HCWs = 19, GP = 17). Most participants (*n* = 364) do not inform themselves about ticks in spring, while only 241 inform themselves about programs for vector control. Nevertheless, almost half of the participants would accept vaccination against TBDs in general (*n* = 286). Interestingly, dog owners show higher adherence to protective practices (F_(1,640)_ = 6.699, *p* = 0.010, η^2^ = 0.010), while the parents of children up to 18 years of age do not (F_(1,640)_ = 0.597, *p* = 0.440).

## 4. Discussion

Tick-borne encephalitis (TBE) poses a growing public health problem in Europe due to its geographical expansion in recent decades [5,10]. The risk of TBE is related to occupational exposure, outdoor leisure activities, and traveling from non-endemic to endemic regions [36]. Estimating knowledge, attitudes, and practices is a quick and easy tool to obtain a rough estimation of people’s awareness regarding ticks and tick-borne diseases [37], as also confirmed by our study.

Our study, based on the comparison of knowledge, attitudes, and practices between three different groups of people (PTEPs, HCWs, and GP), gave an insight into the current state of awareness of TBE and TBDs in Serbia. The estimation of knowledge on TBE and TBDs showed significant differences among these groups. Overall, the knowledge about TBE and TBDs among HCWs and PTEPs is significantly different than that on GP. Expectedly, HCWs are the group with the highest knowledge of TBE and TBDs in our study. Still, critical points in knowledge about TBE and TBDs were identified within all three groups. Similar observations have been made by others. Health care workers play a key role in revealing TBE cases. However, for example, in a study from Poland, the lack of standard operating procedures of TBE diagnosis in Polish hospitals was noted, and the diagnosis of TBE was subjective [38]. In a study from Italy, 58% of occupational physicians had adequate knowledge of TBE, while the accurate perception of TBE risk in occupational settings was found in 20% [39]. We also identified particularly critical questions for which the correct answer rate was low in Serbia, namely questions considering tick biology, TBE etiology, transmission dynamics, and the current epidemiological status in Serbia, and possibilities for immunoprophylaxis. The results might be the consequence of interest in the matter, as well as the availability of education to groups of interest. Similar results of knowledge estimation regarding ticks, TBEV, and TBDs targeting different groups of people in Europe were documented and revealing the need for further education. For example, in France, a study identified the need for further training in serology limitations and the availability of TBE vaccination among primary care physicians [40]. Similarly, only 56.2% of students from Germany, Poland, and Thailand declared their knowledge of the risks posed by ticks and TBPs to be sufficient [41]. Another study from northeastern France underlined the importance of information on TBE among workers exposed to tick bites [42].

While the perception of risk towards TBE and TBDs was generally high in all three groups, it was significantly higher among HCWs and PTEPs. The Health Belief Model, often used in psychological analysis in this field [43,44], highlights that personal experiences and knowledge status are the logical prerequisites to increase risk perception, which in turn promotes behavioral adaptations [39]. The results of risk of ticks perception in our study can also be explained by the level of knowledge on TBE and TBDs among participants [45]. Paradoxically, the fear of ticks, TBE, and TBDs remained low in all three tested groups. Risk related to ticks and TBDs is new to many people, although it can have a potentially serious health impact, the probability of getting a TBD after a tick bite is low, and the risk is difficult to control [46]. This might partially explain, together with the level of knowledge, the lack of fear towards ticks and TBDs in our study. Another possible explanation might be that ticks often provoke a sense of disgust rather than fear [47]. However, the cultural component of fear-to-protective behavior dynamics should not be neglected, as cross-cultural differences in risk perception have been reported for diseases and disasters [48]. Our results may be interpreted as a result of optimistic bias [49]; however, one should be careful in making cross-cultural generalizations, as different tendencies can be observed in other settings.

Observational studies conducted in areas with endemic TBDs have repeatedly found that a large proportion of people fail to take even the most basic of precautions, such as wearing long trousers, using repellent, or avoiding locations of highest risk [50]. The same was true also in our study where only 90 participants declared that they wear white clothes when in nature, while just half of them (*n* = 323) wear long sleeves. The majority check their skin for ticks after being in nature (*n* = 548). This finding is contrary to the results of Mawby and Lovett (1998) when self-reported checking for ticks after potential exposure was low, even though the removal of a tick within 24 h may effectively prevent the transmission of the bacteria and infection [51]. This might also be explained by the fact that, during the time period between the two studies, the awareness of ticks and tick bite prevention increased while the subject was more present in communication media which gave the desired results. Dog owners mostly think that protecting their dog against ticks helps their own protection against ticks (*n* = 376), while the majority (*n* = 441) think that having a dog does not increase the risk of tick acquisition. Furthermore, dog owners are said to be at higher risk of tick bites [52], but this group of participants also shows a higher level of protective measures employed. The same was not true for the people with children, which partially correlates with the result of a study from the USA finding that only parents with a fear of ticks would avoid the outdoors and are more likely to adopt protective measures against ticks [53].

Despite the number of TBE cases per year remaining low in Serbia, participants recognized the possibility of vaccination as a prevention measure in half of the cases through the acceptance of vaccination for TBDs if needed. This fact is encouraging for the future as it has been noted that, especially in some eastern European countries where TBE is endemic and national vaccination campaigns are not in place, vaccine coverage is low and TBE incidence is high. Vaccination, which has not yet been introduced, is a possible approach to the control of TBEV that might soon be adopted in Serbia, keeping in mind the inevitable climatic changes and spreading of areas in which TBEV is endemic.

A psychological theory of behavior change can help increase the success of public education campaigns [30] since it is essential to match behavior change interventions with people’s states of mind. Interventions of this kind may induce shifts in the uptake of a range of personal protective measures. A carefully designed, relatively inexpensive health education message [54] based on social learning theory can be delivered to an at-risk population and result in increased precautionary behavior, as was the case of Lyme borreliosis, even against a background of widespread (mis)information [55]. An objective of public health policies (also in the case of TBE and TBDs) was to induce precautionary behavior without causing alarm for reductions in, for example, outdoor activities [56]. Hence, for the problem of further education on TBDs and TBE, it is crucial to address the right groups of people appropriately through an interdisciplinary approach.

## 5. Conclusions

The assessment of the level of knowledge regarding TBDs and TBE in Serbia, carried out in this study, revealed the weak points where further education is needed. The high perception of the risk of TBDs corresponds to the levels of knowledge and available information. Nevertheless, the fear of TBDs is generally low in Serbia while the implementation of protective measures against ticks and TBDs is still not widely adopted when in nature. These findings will be useful to direct further interventions and the development of a public health strategy against ticks, TBE, and TBDs in Serbia.

## Figures and Tables

**Table 1:** Results of the tested parameters of the three groups of participants in terms of knowledge, attitudes, and practices in Serbia.

Group	Participants (No.)	Mean Age of Participants (Years (CI)/Minimum–Maximum)	Sex (Male/Female/Other)	Having Children under 18 Years	Having Dogs	Tick Bite during the Lifetime	TBDs	TBDs—Family Members
PTEPs	199	42.9 (30.3–55.5)/20–74	101/97/1	72	97	146	8	14
HCWs	147	43.2 (30.6–55.7)/19–75	59/88/0	66	79	82	6	12
GP	296	39.7 (28.4–50.9)/18–76	80/215/1	129	108	165	3	24
Total	642	41.9/18–76	240/400/2	267	284	393	17	50

TBD—tick-borne diseases; PTEPs—professionally tick-exposed persons; HCWs—health care workers; GP—general population.

## Data Availability

The data are available on the MDPI website.

## Supplementary Materials

The following are available online at https://www.mdpi.com/article/10.3390/ijerph19020867/s1, Supplementary Material S1: Questionnaire on the knowledge and attitude towards tick-borne encephalitis and other tick-borne diseases; Supplementary Material S2: The questions on the knowledge and opinion towards ticks and tick-borne diseases and answers of the participants from three different groups.
