# Supplementary material for: Knowledge, Attitudes, and Practices on Tick-Borne Encephalitis Virus and Tick-Borne Diseases within Professionally Tick-Exposed Persons, Health Care Workers, and General Population in Serbia: A Questionnaire-Based Study
Source: Int J Environ Res Public Health. 2022 Jan 13;19(2):867. doi: 10.3390/ijerph19020867 (PMC8775684; doi:10.3390/ijerph19020867)
Supplement: Supplementary file 1 [file ijerph-19-00867-s001.zip › Supplementary Material S1.pdf]

Supplementary Material S1. Questionnaire on the knowledge and attitude of professionally tick exposed persons, health care workers, and the general population, towards tick-borne encephalitis and other tick-borne diseases.

Institute for Medical Research, National Institute of Republic of Serbia, University of Belgrade

Questionnaire on the knowledge and attitude towards tick-borne encephalitis and other tick-borne diseases

We kindly ask you to answer the following questions:

I
Demographic questions

1. How old are you?

	I do not want to answer

2. Gender?

M	F	I do not want to answer

3. Education level (circle around)?

a) Primary school
b) High school
c) Faculty
d) Master
e) Specialization
f) PhD

4. In which city do you currently live?

5. Do you have children under 18 years old?

YES	NO

6. Do you have dog(s) ?

YES	NO

7. Are you a professionally exposed person to tick bites, i.e., are you due to the nature of the job spending time in the natural environment of ticks?

YES	NO

8. Did you experience a tick bite?

YES	NO

9. Did you have one of the tick-borne diseases during your lifetime?

YES	NO

10. Did your family member have a tick-borne disease?

YES	NO

11. Are you a health care worker?

YES	NO

12. If you are a health care worker, are you employed in primary health care?

YES	NO

II
Knowledge on tick-borne encephalitis and tick-borne diseases

1. Did you hear of the term ARBOVIRUS?

YES NO I DO NOT KNOW

2. Did you hear of the term ENDEMIC AREA?

YES NO I DO NOT KNOW

3. Did you hear of tick-borne encephalitis?

YES NO I DO NOT KNOW

4. Do you think that tick-borne diseases are widespread in Serbia?

YES NO I DO NOT KNOW

5. Do you know how many developmental stages ticks have?
YES NO I DO NOT KNOW

6. Do you think that the infected tick can transmit tick-borne encephalitis virus in 12h after the bite?
YES NO I DO NOT KNOW

7. Do you think that tick-borne encephalitis can lead to a lethal cause?
YES NO I DO NOT KNOW

8. Do you think there are registered tick-borne encephalitis cases in Serbia?
YES NO I DO NOT KNOW

9. Do you know is there a vaccine against tick-borne encephalitis?
YES NO I DO NOT KNOW

10. Do you think that tick-borne encephalitis is widespread in Europe?

YES NO I DO NOT KNOW

11. Do ticks transmit causative agents of Lyme borreliosis?

YES NO I DO NOT KNOW

12. Do ticks transmit the West Nile virus?

YES NO I DO NOT KNOW

13. Do ticks transmit Crimean-Congo hemorrhagic fever virus?

YES NO I DO NOT KNOW

14. Do you think that only one tick species feed on humans?

YES NO I DO NOT KNOW

15. Attached tick is being extracted using medicine petrol by rules?

YES NO I DO NOT KNOW

16. Attached tick is being extracted using tweezers by rules?

YES NO I DO NOT KNOW

17. Attached tick should be extracted in whole?

YES NO I DO NOT KNOW

18. Is each tick infected with some of the tick-borne pathogens?

YES NO I DO NOT KNOW

19. Is the causative agent of tick-borne encephalitis a bacterium?

YES NO I DO NOT KNOW

III
Risk and fear perception

On a scale from 1 to 5 (1 I strongly disagree, 3 I do not know if I agree or disagree, 5 I strongly agree) circle the answer which fits best to your attitude.

1. I think I enter into risky situations in everyday life

I strongly disagree I strongly agree

1 2 3 4 5

2. Stay in nature for 15 minutes is a risk for the tick bite.

1 2 3 4 5

3. Prolonged stay in nature is a higher risk for the tick bite than a short stay.

1 2 3 4 5

4. Tick bite can lead to the development of a tick-borne disease.

1 2 3 4 5

5. Tick-borne encephalitis can lead to a lethal outcome.

1 2 3 4 5

6. Risk of tick-borne encephalitis is raising in Europe.

1 2 3 4 5

7. Vaccination can protect against tick-borne encephalitis.

1 2 3 4 5

8. Field walk is a risk for a tick bite.

1 2 3 4 5

9. Walk in the woods is a risk for a tick bite.

1 2 3 4 5

10. Walk near the riverbank is a risk for a tick bite.

1 2 3 4 5

11. Walk on the signed pathway is a risk for a tick bite.

1 2 3 4 5

12. Tick can pass from my dog to me.

1 2 3 4 5

13. Tick bite is a significant risk for health.

1 2 3 4 5

14. I am afraid of ticks.

1 2 3 4 5

15. I am afraid of getting a tick-borne disease.

1 2 3 4 5

16. Fear of tick bite keeps me away from the green-grass fields.

1 2 3 4 5

17. Fear of tick bite changed my wish to stay in nature.
1 2 3 4 5

18. Because of the fear of tick bites I do not allow children to play in the grass.
1 2 3 4 5

19. Fear of the ticks affects my everyday life.

1 2 3 4 5

20. Fear of tick-borne encephalitis affects my everyday life.

1 2 3 4 5

IV

Knowledge on protection from the tick bite

1. Repellents (spray, cream, tick bite protection) protect against ticks.

YES NO I DO NOT KNOW

2. I wear a long-sleeve wardrobe for protection against ticks.

YES NO I DO NOT KNOW

3. I wear a white wardrobe for protection against ticks while in nature

YES NO I DO NOT KNOW

4. I wear white socks over the pants for protection from ticks

YES NO I DO NOT KNOW

5. I check my skin after a stay in nature

YES NO I DO NOT KNOW

6. Regular protection against ticks of my dog helps my protection against ticks

YES NO I DO NOT KNOW

7. Vaccination against tick-borne encephalitis can help my protection against the disease.

YES NO I DO NOT KNOW

8. I was tested for antibodies against tick-borne diseases.

YES NO I DO NOT KNOW

9. Before traveling abroad I inform myself about the diseases present in the destination country.

YES NO I DO NOT KNOW

10. I asked my physician for some advice on necessary vaccinations before traveling abroad.

YES NO I DO NOT KNOW

11. I asked my physician about the protection against ticks in my workplace.

YES NO I DO NOT KNOW

12. In general, I would accept vaccination against tick-borne diseases.

YES NO I DO NOT KNOW

13. I avoid high grass to protect myself from the tick bite.

YES NO I DO NOT KNOW

14. I inform myself about tick-borne diseases in spring.

YES NO I DO NOT KNOW

15. I follow information about tick-borne diseases in general.

YES NO I DO NOT KNOW

16. I am informed about vector control programs in public green areas.

YES NO I DO NOT KNOW

17. Having a dog increases the risk of a tick bite.

YES NO I DO NOT KNOW
