# Supplementary material for: Knowledge, Attitudes, and Practices on Tick-Borne Encephalitis Virus and Tick-Borne Diseases within Professionally Tick-Exposed Persons, Health Care Workers, and General Population in Serbia: A Questionnaire-Based Study
Source: Int J Environ Res Public Health. 2022 Jan 13;19(2):867. doi: 10.3390/ijerph19020867 (PMC8775684; doi:10.3390/ijerph19020867)
Supplement: Supplementary file 1 [file ijerph-19-00867-s001.zip › Supplementary Material S2.pdf]

Supplementary Material S2. The questions on the knowledge and opinion towards ticks and tick-borne diseases and answers of the participants from three different groups.

Question	PTEP correct answers	HCW correct answers	GP correct answers	Total (positive answers; %; CI)	Group differences F(df=2), p
Did you hear for the term ARBOVIRUS?	123	120	100	343 (53.4) [49.5-57.3]	57.917, < 0.001
Did you hear for the term ENDEMIC AREA?	190	145	275	610 (95.0) [93.0-96.6]	3.496, 0.031
Did you hear for TBE?	164	138	188	490 (76.3) [72.8-79.6]	30.550, < 0.001
Do you think that TBDs are widespread in Serbia?	168	116	190	474 (73.8) [70.2-77.2]	14.440, < 0.001
Do you know how many developmental stages ticks have?	124	86	31	241 (46.9) [43.0-50.8]	31.704, < 0.001
Do you think that the infected tick can transmit TBEV in 12h after the bite?	91	84	120	395 (46.0) [42.0-49.9]	5.522, 0.004
Do you think that TBE can lead to the lethal cause?	139	130	174	443 (69.0) [65.3-72.6]	21.496, < 0.001
Do you think there are registered TBE cases in Serbia?	97	92	136	325 (50.6) [46.7-54.6]	5.717, 0.003
Do you know is there a vaccine against tick-borne encephalitis?	31	42	39	112 (17.4) [14.6-20.6]	8.619, < 0.001
Do you think that TBE is widespread in Europe?	105	79	128	312 (48.6) [44.7-52.5]	3.185, 0.042
Do ticks transmit causative agents of Lyme borreliosis?	191	144	261	596 (92.8) [90.6-94.7]	9.439, < 0.001
Do ticks transmit WNV?	119	96	172	387 (60.3) [56.4-64.1]	1.075, 0.342
Do ticks transmit CCHFV?	41	50	31	122 (19.0) [16.0-22.3]	18.892, < 0.001

Do you think that only one tick species feed on humans?	169	130	197	496 77.3 [73.8-80.4]	19.211, < 0.001
Attached tick is being extracted using medical petrol by rules?	138	112	148	398 (62.0) [58.1-65.8]	18.541, < 0.001
Attached tick is being extracted using tweezers by rules?	150	114	190	454 (70.7) [67.0-74.2]	5.824, 0.003
An attached tick should be extracted in whole?	192	144	292	628 (97.8) [96.4-98.8]	1.317, 0.269
Is each tick infected with some of the TBPs?	176	135	223	534 (83.2) [80.1-86.0]	12.849, < 0.001
Is the causative agent of TBE a bacterium?	28	32	51	111 (17.3%) [14.4-20.4]	1.754, 0.174

TBE-tick-borne encephalitis, TBDs-tick-borne diseases, TBEV-tick-borne encephalitis virus, WNV-West Nile virus, CCHFV-Crimean Congo hemorrhagic fever virus, TBPs tick-borne pathogens, PTEP-professionally tick-exposed persons, HCW-health care workers, GP-general population.
